# Beyond germline genetic testing - heterozygous pathogenic variants in *PMS2* in two children with Osteosarcoma and Ependymoma

**DOI:** 10.1186/s13053-023-00254-4

**Published:** 2023-06-12

**Authors:** Michaela Kuhlen, Mariola Monika Golas, Tina Schaller, Nicole Stadler, Felicitas Maier, Olaf Witt, Michael C. Frühwald

**Affiliations:** 1 Pediatrics and Adolescent Medicine, Faculty of Medicine, University of Augsburg, Augsburg, Germany; 2 Swabian Children´s Cancer Center, University Medical Center Augsburg, Stenglinstr. 2, 86156 Augsburg, Germany; 3 Human Genetics, Faculty of Medicine, University of Augsburg, Augsburg, Germany; 4 Department of Hematology and Medical Oncology, Comprehensive Cancer Center Augsburg, University Medical Center Augsburg, Augsburg, Germany; 5 Pathology, Faculty of Medicine, University of Augsburg, Augsburg, Germany; 6 Center for Human Genetics and Laboratory Medicine Martinsried, Germany, and Medical Practice for Genetic Counselling and Psychotherapy, Augsburg, Germany; 7 German Cancer Research Center (DKFZ), Hopp Children’s Cancer Center Heidelberg (KiTZ), Heidelberg University Hospital, Heidelberg, Germany

**Keywords:** Hereditary cancer predisposition, *PMS2*, Children, Osteosarcoma, Ependymoma, Lynch syndrome

## Abstract

**Background:**

Lynch syndrome (LS) is not considered part of childhood cancer predisposition syndromes.

**Case presentation:**

Analysis of a pediatric osteosarcoma (OS) displayed hypermutation (16.8), alternative lengthening of telomeres (ALT), loss of PMS2 expression in tumor tissue (retained in non-neoplastic cells), *PMS2* loss of heterozygosity (LOH), and high-degree of microsatellite instability (MSI) tested by PCR. A heterozygous duplication c.1076dup p.(Leu359Phefs*6) in exon 10 of NM_000535.6:*PMS2* was detected by SNV analysis in peripheral blood, confirming diagnosis of LS in the patient. The tumor molecular features suggest LS-associated development of OS. In a second case, whole-genome sequencing identified a heterozygous SNV c.1 A > T p.? in exon 1 of *PMS2* in tumor and germline material of a girl with ependymoma. Tumor analysis displayed evidence for ALT and low mutational burden (0.6), PMS2 expression was retained, MSI was low. Multiplex ligation-dependent probe amplification identified no additional *PMS2* variant and germline MSI testing did not reveal increased gMSI ratios in the patient´s lymphocytes. Thus, CMMRD was most closely excluded and our data do not suggest that ependymoma was related to LS in the child.

**Conclusions:**

Our data suggest that the LS cancer spectrum may include childhood cancer. The importance of LS in pediatric cancers necessitates prospective data collection. Comprehensive molecular workup of tumor samples is necessary to explore the causal role of germline genetic variants.

**Supplementary Information:**

The online version contains supplementary material available at 10.1186/s13053-023-00254-4.

## Background

The awareness of genetic predisposition in children and adolescents affected by cancer is steadily increasing. Cancer predisposition syndromes (CPS) have been reported in up to 8.5% of cases [1, 2]. CPS are associated with a range of pediatric malignancies. Vice versa typical pediatric cancers are associated with a number of CPS [3]. Likely, these associations will increase in numbers over coming years.

Comprehensive genetic testing of tumor samples included in the diagnosis of cancer may indicate an underlying genetic predisposition due to a high number of mutational events, specific patterns and signatures of somatic mutations (including near-heterozygous and near-homozygous allelic frequency), and chromothripsis, amongst others. Here, we report two females, in whom comprehensive tumor analyses of relapsed osteosarcoma and ependymoma, respectively, exposed evidence for an underlying genetic predisposition.

Osteosarcoma (OS) is the most frequent bone tumor in children and adolescents. Approximately 10% of patients harbor germline pathogenic variants in *TP53* [1, 4]. OS, however, occurs in different CPS including Li-Fraumeni syndrome, retinoblastoma predisposition, and Rothmund-Thomson syndrome among others (reviewed in [3]). A recent study among 1,244 patients with OS (mean age at diagnosis, 16 years) indicated that 28% of patients harbored pathogenic (P) or likely pathogenic (LP) cancer susceptibility gene variants in *TP53* and in genes previously not linked to OS (e.g., *CDKN2A*, *MEN1*, *VHL*, *POT1*, *APC*, *MSH2*, *ATRX*) [5]. Another study comprising 1,120 children and adolescents with various types of pediatric cancers identified germline P/LP variants in 7 of 39 (18%) OS patients [1].

Ependymoma is a rare type of primary brain or spinal cord tumors, accounting for approximately 10% of all central nervous system tumors in children and adolescents. The genetic predisposition landscape in children with ependymoma is still poorly understood. Whole genome and exome analysis in 1,120 children and adolescents identified germline P/LP variants in *NF1*, *NF2* and *TP53* in 6% of 67 ependymoma patients [1]. In contrast, pan-cancer analyses in 961 tumors from children and adolescents did not identify any germline P/LP variant in 59 patients with ependymoma [2]. In case reports of patients with multiple ependymomas, germline P/LP variants in *APC* were reported. [6, 7]

## Case presentation

### Case report 1 (CR1)

**Fig. 1:**
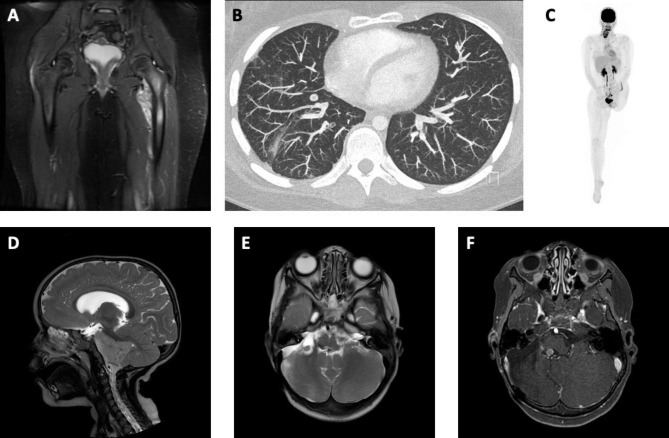
Magnetic resonance imaging (MRI), computed tomography (CT) and positron-emission tomography (PET). CR1: **A** Coronal T2-weighted MRI of the femur and pelvic region demonstrating the tumor of the left proximal femur. **B** CT scan demonstrating a single pulmonary metastasis. **C** PET scan demonstrating a large metastasis at the back and multiple cervical und nuchal metastases. CR2: **D** Sagittal T2-weighted MRI demonstrating the tumor of the posterior fossa encircling the brain stem. **E** Axial T2-weighted MRI demonstrating first local relapse. **F** Axial T2-weighted-Fluid-Attenuated MRI demonstrating second local relapse

Following a four-month period of pain, the 12-year-old girl was diagnosed with OS of the proximal femur and an extensive intravascular tumor thrombus extending into the external iliac and pulmonary veins (Fig. 1A). Treatment according to an osteosarcoma protocol (EURAMOS 1/COSS) with six courses of MAP chemotherapy (methotrexate, adriamycin, cisplatin) was administered. Tumor resection including MUTARS® endoprosthesis for reconstruction was performed after two cycles. Regression stage according to Salzer-Kuntschik was classified as grade 3. Surgical margins were deemed to be insufficient in the area of the deep iliac veins. Hence, external hemipelvectomy was performed subsequently. On suspicion of pulmonary tumor emboli in the upper and lower lobe, atypical resections were performed after completion of standard chemotherapy. Histological evaluation confirmed avital metastases of OS. At the age of 15 years, a single pulmonary metastasis was diagnosed (Fig. 1B). Following segmentectomy the patient was administered 36 weeks of immune stimulatory therapy with mifamurtide. At the age of 17 years, the patient presented with a large intramuscular metastasis in the erector spinae and cervical and nuchal lymph node metastases (Fig. 1C). Partial resection of the muscle including replacement and lateral neck dissection were performed. The patient remains in complete radiological remission three years later.

To identify therapeutic targets, tumor analysis of the second relapse was conducted within the framework of the INFORM project [8] including whole-exome, low-coverage whole-genome, RNA sequencing, and methylation as well as expression microarray analyses. Analyses of the nuchal metastasis displayed increased genomic instability with hypermutation (tumor mutational burden 16.8) and alternative lengthening of telomeres (ALT) (Table 1). Accordingly, PD1/PDL1 and PARP inhibitors constituted potential therapeutic options.

**Fig. 2:**
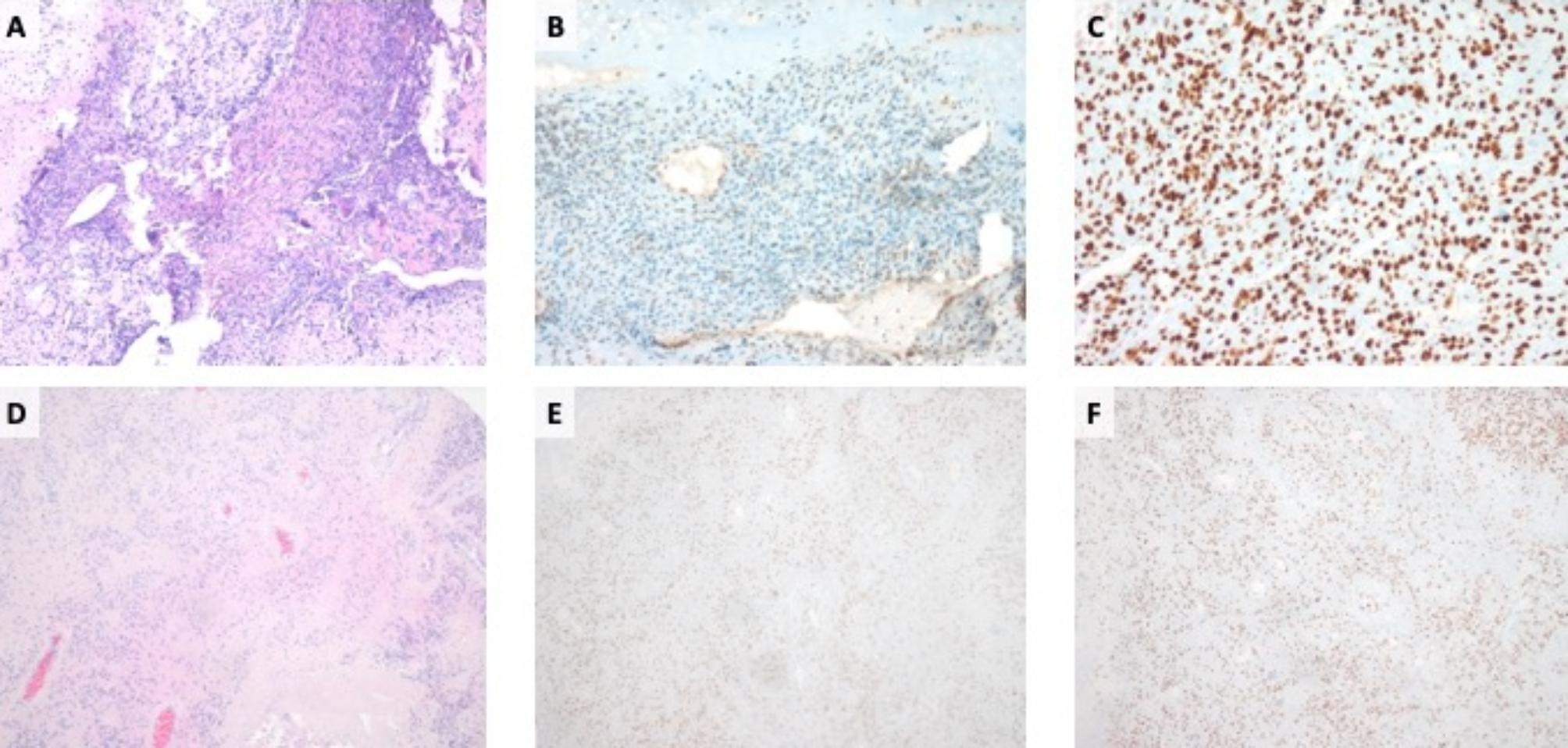
CR1: (**A)** Hematoxylin and eosin staining of the nuchal metastasis of the osteosarcoma. Immunohistochemical (IHC) staining of mismatch repair proteins demonstrating loss of PMS2 expression in **(B)** neoplastic cells and **(C)** retained nuclear expression of MSH6 in the tumor cells. CR2: (**D**) Hematoxylin and eosin staining of the 2nd relapse of ependymoma. IHC of mismatch repair proteins demonstrating nuclear expression of PMS2 (**E**) and MSH6 (**F**) in the tumor cells. A-B, D-F: 40x magnification. C: 100x magnification

Based on the genetic analyses, suspicion of a mismatch repair defect was raised and immunohistochemical (IHC) staining of mismatch repair (MMR) proteins and microsatellite instability (MSI) analyses were prompted. Loss of PMS2 expression was seen in tumor tissue, while PMS2 expression in non-neoplastic cells was retained (Fig. 2A-C). A high-degree of MSI (MSI-H) tested by PCR was observed. In line with this, loss of heterozygosity (LOH) of *PMS2* in tumor tissue was demonstrated by the INFORM analyses. To further elucidate genetic predisposition, peripheral blood of the patient was analyzed via NGS sequencing for single nucleotide variants (SNVs).

A heterozygous duplication c.1076dup p.(Leu359Phefs*6) in exon 10 of NM_000535.6: *PMS2* was detected by SNV analysis and confirmed by Sanger sequencing. Based on the classification criteria developed by the InSIGHT Variant Interpretation Committee for MMR gene variants v2.4 (2018-06), the *PMS2* variant was classified as pathogenic (class 5; see also Supplemental Information), confirming the patient´s diagnosis of LS.

Daily use of ASA was recommended to the patient and LS surveillance initiated.

The family history in the three preceding generations was remarkable for renal cancer in the grandmother (deceased at the age of about 60 years) and leukemia in a grandaunt (deceased at the age of 50 years) in the paternal line. Complementary analysis of the parents´ blood confirmed that the *PMS2* variant was inherited by the thus far clinically unaffected father. LS surveillance was recommended to the father. The maternal line was unsuspicious in terms of cancer.

### Case report 2 (CR2)

The one-year-old girl presented with torticollis and an anamnestic episode of a tonic-clonic convulsive seizure a few days before admission. Ophthalmological examination revealed evidence of Heimann-Bielschowsky phenomenon suspicious of trochlear nerve palsy. Magnetic resonance imaging (MRI) demonstrated a posterior fossa tumor encircling the brain stem, growing into the internal acoustic meatus inducing occlusive hydrocephalus (Fig. 1D). Metastatic disease of the craniospinal axis or the cerebrospinal fluid was excluded. Total tumor resection was performed and histological evaluation showed anaplastic ependymoma WHO°III/posterior fossa ependymoma methylation subgroup A. Concurring with current international data and considering the patient´s age, proton beam therapy at a dose of 54 Gy was administered. At the age of 2.7 years, local relapse was diagnosed (Fig. 1E). Following tumor resection, proton beam therapy with a dose of 54 Gy was administered. At the age of 4.3 years, follow-up MRI demonstrated 2nd local relapse. Once more a tumor resection was performed. Due to radiotherapy-related occlusion of both carotid arteries, the patient subsequently underwent angioplasty and since then is on acetylsalicylic acid (ASA) therapy. Three years later, the patient sustained a 3rd local relapse of ependymoma.

Analyses of the 2nd relapse within the INFORM project did not identify therapeutic targets. DNA methylation-based classification confirmed a group A posterior fossa ependymoma (PF-A). It further displayed evidence for ALT and low tumor mutational burden (0.6). Whole-genome sequencing (WGS) analysis, however, identified the heterozygous SNV c.1 A > T p.? in exon 1 of *PMS2* in tumor and germline material and was further confirmed by Sanger sequencing in peripheral blood of the patient. According to the InSIGHT criteria this variant is classified as likely pathogenic (class 4; see supplement).

The family history was remarkable for acute myeloid leukemia (not further specified) in the mother in young adulthood and a cousin on the maternal side who deceased of sarcoma (not further specified) at the age of 5 years. The family history on the paternal side was unsuspicious. The parents as yet refused genetic testing of themselves.

To further elucidate the role of the *PMS2* germline variant in tumor development, we initiated IHC and MSI analysis. In tumor material of the 2nd relapse, PMS2 expression was retained, and MSI was low (Fig. 2D-F). To test for a second pathogenic variant in *PMS2* in terms of constitutional mismatch repair deficiency (CMMRD), multiplex ligation-dependent probe amplification (MLPA) was prompted and identified no additional *PMS2* variant. In addition, germline MSI (gMSI) testing [9] did not reveal increased gMSI ratios in the patient´s lymphocytes and, hence, could not confirm CMMRD in the child.

**Table 1:** Details on patients´ characteristics and diagnostics

Characteristics	Patient CR1	Patient CR2
Age/Gender	12 y/female	1 y/female
Cancer	Osteosarcoma	Anaplastic ependymoma WHO °III/posterior fossa ependymoma methylation subgroup A
Family history	- Paternal grandmother: renal cancer, died at about 60 years of age - Paternal grandaunt: leukemia, died at 50 years of age - Father: carrier of *PMS2* variant - Parents non-consanguinous	- Mother: AML young adulthood - Maternal cousin: sarcoma, died aged 5 years - Parents: no carrier testing - Parents non-consanguinous
MMR IHC (metastasis)	Loss of PMS2 expression in tumor tissue	Retained PMS2 expression
MSI (metastasis)	MSI-H	MSI-L
Other genetic results	- Hypermutation (tumor mutational burden 16.8) - Alternative lengthening of telomeres - LOH of *PMS2* in tumor tissue	- Tumor mutational burden 0.6 - Alternative lengthening of telomeres - No gMSI in peripheral blood
Sequencing (peripheral blood)	*PMS2*: c.1076dup p.(Leu359Phefs*6) heterozygous (class 5 according to the InSIGHT criteria)	*PMS2*: c.1 A > T p.? heterozygous (class 4 according to the InSIGHT criteria)

## Discussion and conclusions

In both patients, pathogenic *PMS2* variants were identified by peripheral blood analysis via NGS highly suggestive of diagnosis of LS. LS is caused by heterozygous germline P/LP variant in one of the four DNA MMR genes *MLH1*, *MSH2*, *MSH6*, and *PMS2* or *EPCAM* deletions. [10] LS is associated with an increased risk of colorectal and gynecological cancers manifesting during the fourth and fifth decade of life in average, but other cancers such as urinary tract and gastric cancers, among other tumors, may also arise [11].

Sarcomas are not considered part of the commonly observed LS tumor spectrum. The Pediatric Cancer Genome Project, however, reported 2 of 39 patients (5.1%) with OS with P/LP heterozygous *MSH2* germline variants. In the pan-cancer analysis of various pediatric cancers, one patient with OS carrying a P/LP *MSH2* variant was identified [1, 2]. In a meta-analysis of 11 studies incorporating comprehensive germline testing for children and adolescents with cancer, a P/LP germline variant in one of the MMR genes was reported in 19 of 3975 patients including 9 patients with *PMS2* variants (brain tumors n = 4, nonbrain solid tumors n = 4, hematologic neoplasms n = 1) [12]. In an international genetic study exploring 1,162 patients with sarcoma, 11 patients carried P/LP germline variants in MMR genes [13]. A study assessing patients with LS and sarcoma reported 27 of 178 patients (15.2%) with sarcomas in the same individual or families. In two *MSH2* carriers with OS, the sarcomas were confirmed as LS-related since the tumors were MSH2/MSH6-deficient and MSI-H [14]. A recent study reported among 1,244 patients with OS a higher-than-expected frequency of P/LP variants in *MSH2* [5]. In this study, carriers of a P/LP *PMS2* variant were reported only occasionally. This is in accordance with the frequency of P/LP *PMS2* variants in LS patients of less than 5 to 8%. Of note, osteosarcoma was recently reported in two patients with CMMRD with compound heterozygous variants in *PMS2* [15]. In the present study, loss of PMS2 expression and MSI-H in tumor tissue of patient CR1 were determined, while non-neoplastic cells were immunopositive for PMS2. Consistent with these observations, a pathogenic germline variant in *PMS2* coupled to LOH of *PMS2* in tumor tissue was identified in the patient. Although we cannot rule out the possibility of a second germline P/LP variant in PMS2, which may have escaped detection, the LOH of *PMS2* in the OS together with the retained PMS2 expression in non-neoplastic cells strongly supports the diagnosis of LS rather than CMMRD in patient CR1.

Genes involved in DNA repair pathways including MMR have previously been associated with pediatric brain tumor susceptibility. [12] While CMMRD is commonly associated with the development of brain tumors, the occurrence of brain tumors in LS patients is less common. In 1996, an analysis from the Dutch HNPPC registry reported one patient with ependymoma and LS [16]. The two largest studies on germline variants in pediatric cancers, on the other hand, did not identify any patient with ependymoma carrying a P/LP MMR gene variant [1, 2]. A recent study from the European C4CMMRD consortium reporting 87 patients with CMMRD including 56 brain tumors did not report any patient with ependymoma [15]. Of 49 patients with brain tumors in this series, however, 27 harbored homozygous or compound heterozygous variants in *PMS2*. Only recently, the first case of ependymoma in a young child with CMMRD was reported. [17] On the other hand, two large studies on cancer risks in LS patients did not find an increased risk for brain tumors in heterozygous *PMS2* variant carriers [18, 19]. In line with this, our data do not suggest that ependymoma was related to LS in CR2. It thereby demonstrates that screening for mutations reveals only part of the story and comprehensive assessment of a tumor profile is necessary to explore both therapeutic targets as well as a potential genetic predisposition.

## Conclusion

Along with previous reports, our data link a typical pediatric cancer, OS, with LS. The role of LS in pediatric cancers, however, is still poorly understood necessitating prospective data collection.

Comprehensive molecular workup of tumor samples is necessary to explore the causal role of germline genetic variants.

## Electronic supplementary material

Below is the link to the electronic supplementary material.

Supplementary Material 1

## Data Availability

Data are available upon reasonable request.

## Abbreviations

ALT: Alternative lengthening of telomeres
ASA: Acetylsalicylic acid
CMMRD: Constitutional mismatch repair deficiency
CPS: Cancer predisposition syndrome
CR: Case report
IHC: Immunohistochemical
LOH: Loss of heterozygosity
LS: Lynch syndrome
MMR: Mismatch repair
MRI: Magnetic resonance imaging
(g)MSI: (Germline) Microsatellite instability
OS: Osteosarcoma
P/LP: Pathogenic/likely pathogenic
