# Supplementary material for: Beyond germline genetic testing - heterozygous pathogenic variants in PMS2 in two children with Osteosarcoma and Ependymoma
Source: Hered Cancer Clin Pract. 2023 Jun 12;21:8. doi: 10.1186/s13053-023-00254-4 (PMC10259054; doi:10.1186/s13053-023-00254-4)
Supplement: Supplementary file 1 — Supplementary Material 1 [file 13053_2023_254_MOESM1_ESM.docx]

**Supplementary information**

**Methods**

For germline sequencing and DNA extraction, EDTA peripheral blood sample was obtained. Extracted DNA was enriched (Agilent Sure Select) and next generation sequencing (Illumina) including coding exons and conserved areas of the splice regions was performed. Data was analyzed using SeqPilot. For the confirmation analysis a second, independently isolated DNA isolation and data analysis using SeqPilot was performed. Gene dose was determined from NGS data with ExomeDepth, XHMM, and CoNVaDING. Hg19 NCBI GRCh37 served as reference genome. Quality criteria were determined as the following: Sequencing runs with quality score> 30 (accuracy> 99.9%) for at least 75% of the bases; sequence analysis of areas with a coverage <20-fold using Next Generation Sequencing (NGS) (Ion Torrent PGM).

**Table S1:** Classification of *PMS2* variants according to InSIGHT and ACMG criteria

|  | *PMS2*:  c.1076dup p.(Leu359Phefs*6) | *PMS2*: c.1A>T p.? |
| --- | --- | --- |
| InSIGHT criteria | - Frameshift alteration in exon 10 of 15   **=> pathogenic (class 5)** | - variant in the initiation codon of *PMS2*  **=> likely pathogenic (class 4)** |
| ACMG criteria | - PVS1 (null variant) - PM2 absent from controls - PP4 phenotype/family history highly specific for a disease   **=> pathogenic (class 5)** | - PVS1 (null variant)  - PM2 absent from controls  **=> likely pathogenic (class 4)** |
